# Evolutionary and functional implications of 3′ untranslated region length of mRNAs by comprehensive investigation among four taxonomically diverse metazoan species

**DOI:** 10.1007/s13258-019-00808-8

**Published:** 2019-03-21

**Authors:** Wei Wang, Dong-hui Fang, Jia Gan, Yi Shi, Hui Tang, Huai Wang, Mao-zhong Fu, Jun Yi

**Affiliations:** Animal Breeding and Genetics Key Laboratory of Sichuan Province, Sichuan Animal Science Academy, Chengdu, 610066 Sichuan People’s Republic of China

**Keywords:** 3′ untranslated regions, Eukaryotic mRNAs, Sequence length, Functional implications

## Abstract

**Background:**

In eukaryotic organisms, it has been well acknowledged that 3′ untranslated regions (3′ UTRs) of mRNA are actively involved in post-transcriptional regulations of gene expression. Although both shortening and lengthening of 3′ UTRs of specific candidate genes were explicitly documented to have functional consequences, landscape of 3′ UTR lengths in relation to evolutionary dynamics and biological meanings remains to be elucidated when large-scale data become available.

**Objectives:**

The primary objective of this study was to revealed different inter- and intra-species patterns on length distribution of 3′ UTRs in comparison with 5′ UTRs and coding regions.

**Methods:**

In the present study, we investigated 3′ UTR lengths in a highly curated set of 57,135 mRNA sequences among four well-studied and taxonomically diverse metazoan species (fruit fly, zebrafish, mouse and human).

**Results:**

The average length ratio of 3′–5′ UTRs considerably increased from fruit fly (twofold) to human (fivefold). Moreover, genes can be characterized by the obviously different patterns of evolutionary change on 3′ UTR lengths. By utilizing the Gene Ontology annotations, genes with differential lengths of 3′ UTRs were suggested to have the divergent functional implications. In human, we further revealed that ubiquitously transcribed genes had higher median lengths of 3′ UTRs than the genes of tissue-restricted expressions.

**Conclusion:**

We conducted a comprehensive analysis and provided an overview regarding 3′ UTRs biology of mRNAs in animals, whereas the mechanistic explanations of 3′ UTRs length variation in correlation to regulation of gene expression still remain to be further studied.

**Electronic supplementary material:**

The online version of this article (10.1007/s13258-019-00808-8) contains supplementary material, which is available to authorized users.

## Introduction

The increased complexity of both morphology and biological functions in higher eukaryotes must be accompanied by a fine-tuning regulation mechanism at the molecular level. For eukaryotic genes, it has long been acknowledged that both 5′ and 3′ untranslated regions (UTRs) of mRNA are significantly involved in post-transcriptional regulation of gene expression, including the modulations of nucleo-cytoplasmic transport, degradation rate, subcellular localization and translation efficiency of mRNA molecules (Mortimer et al. 2014). The alterations in features of mRNA UTRs, such as the sequence length, secondary structures, and composition of both *cis*-acting elements and microRNA binding motifs, have been widely reported to have functional consequences (Chatterjee and Pal 2009; Matoulkova et al. 2012; Wachter 2014). The considerable variation in length observed for 3′ UTRs may suggest that it is a more active player for functional regulations than 5′ UTRs (Chatterjee and Pal 2009).

Recently, the high-throughput sequencing approaches, especially for technique of poly(A)-position profiling by sequencing, were successfully applied to genome-wide profiling of 3′ UTRs landscape in *Caenorhabditis elegans* and reported that most of protein-coding genes are characterized by alternative isoforms of 3′ UTRs (Mangone et al. 2010; Jan et al. 2011). Moreover, both sequence length and expression level contribute to the overall complexity of 3′ UTR isoforms. A specific bioinformatic algorithm was also proposed to quantify 3′ UTR usages based on RNA-seq data, which would be useful for studying functional and regulatory implications of 3′ UTRs (Kim et al. 2015). Within 3′ UTRs, there are a variety of canonical *cis*-acting regulatory elements, such as the adenylate uridylate-rich elements and selenocysteine insertion sequence elements; and all of these elements will be specifically recognized and bound by *trans* binding factors (Szostak and Gebauer 2013). Additionally, 3′ UTRs are intensively studied because of extensive presences of microRNA binding sites (Lytle et al. 2007). Therefore, a method of in silico prediction has been designed for comparing the sequence features of 3′ UTRs (Biswas and Brown 2014).

The alterations of 3′ UTR lengths, especially for shortening events, have been documented to be prevalent under pathologic conditions (Akman et al. 2015; De et al. 2016). In addition to structural and compositional features, therefore, the evolutionary dynamics of 3′ UTR lengths has been also addressed in eukaryotic mRNAs. The median length of 3′ UTRs in mammals is about six times as long as *C. elegans*, whereas the latter has higher density of microRNA binding sites (Jan et al. 2011). Despite this, the absolute number of microRNA binding sites was observed to increase along the lengthening of 3′ UTRs within and across metazoan species (Chen et al. 2012). Liu and colleagues (Liu et al. 2012) analyzed UTR sequences from three eukaryote kingdoms and reported that the length of 3′ UTRs was positively correlated with the number of tandem repeat sequences instead of genome size. By contrast, an exponential relationship between median length of 3′ UTRs and morphological complexity was observed in multiple metazoan species, which suggested the lengthening of 3′ UTRs would be driven by natural selection (Chen et al. 2012).

Due to advances of high-throughput sequencing technologies, massive amounts of gene sequences can be publicly available for intensive mining. For example, the RefSeq project at National Center for Biotechnology Information (NCBI) maintains a large-scale database of genome, transcript and protein reference sequences with the expert-curated quality (O’Leary et al. 2015). In the present study, we utilized RefSeq database and comprehensively investigated the 3′ UTR lengths of mRNAs among four well-studied and taxonomically diverse metazoan species, for which the sufficient and reliable reference gene sequences can be available now. The results would provide more insights into 3′ UTR biology of mRNA in animals.

## Materials and methods

### Retrieval and processing of mRNA sequences

After a preliminary check on NCBI RefSeq database (Release 71), we found that sufficient reference sequences (more than 10,000 non-redundant records) with high reliability are only available for three well-studied metazoan model species (fruit fly, zebrafish and mouse) and human. Subsequently, we retrieved all mRNA records for the four species using the ACNUC Sequence Retrieval System (Gouy and Delmotte 2008). To guarantee high reliability, only manually curated mRNA sequences with accession prefixes of ‘NM_’ were retained. After this, we filtered out these sequences which were already annotated with the labels of ‘incomplete’ on 3′ and/or 5′ ends; and this filter, however, can’t absolutely ensure that all our analyzed mRNA sequences are fully complete. Moreover, to avoid the potential bias for calculating the length ratio of 3′–5′ UTRs, the sequences with 5′ UTR lengths shorter than 30 bp or 3′ UTR lengths shorter than 50 bp were also discarded. Finally, we selected the longest transcript as representative sequence for each gene because all of them have been manually curated and then fed to these following analyses. The numbers of retrieved and processed mRNA sequences among the four species were shown in Table 1; and for each of them the lengths of 5′ UTR, coding region, and 3′ UTR were recorded.

**Table 1:** Length and range of 3′ UTRs among various gene sets

Species	Length	Length-based categorization (bp)				Random categorization (bp)			
		Q1	Q2	Q3	Q4	R1	R2	R3	R4
Human	Mean	220	642	1428	3173	964	1002	986	933
	Min	50	404	974	2058	50	50	50	50
	Max	404	974	2058	24,506	13,669	17,893	13,761	24,506
Mouse	Mean	183	552	1238	2692	860	854	831	850
	Min	50	338	848	1782	50	50	50	50
	Max	338	848	1781	15,948	11,756	15,948	15,587	14,333
Zebrafish	Mean	156	364	650	1256	489	494	492	496
	Min	50	59	493	868	50	52	50	50
	Max	259	493	868	7622	6083	5052	7622	6793
Fruit fly	Mean	87	182	421	1270	284	277	263	259
	Min	50	126	273	679	50	50	50	50
	Max	126	273	679	18,495	11,538	8558	12,542	9011

### Identification of orthologous genes and clustering

To investigate gene-wise variations of 3′ UTR lengths from fruit fly to human, we first determined the orthologous relationship for all analyzed genes in the present study across fruit fly, zebrafish, mouse and human. A total of 2576 orthologous groups among the four species were finally determined according to annotations of OrthoDB v8 (Kriventseva et al. 2015). Within each group, if multiple genes from one species were equally determined as orthologues, we calculated and adopted the mean length of 3′ UTRs for this species. Herein, each orthologous gene has four values of 3′ UTR lengths among fruit fly, zebrafish, mouse and human in order. Subsequently, it is feasible to cluster all genes into different groups according to their variation patterns of 3′ UTR lengths, such as the continuous increase or decrease, from fruit fly to human. Finally, we employed the Euclidean distance measure and k-mean algorithm for clustering genes using the R package Cluster.

### Functional annotation and gene set-based comparison

In the present study, we designed a straightforward strategy to test whether 3′ UTR lengths correlate with the differential gene functions. First, the annotated GO terms for each gene were directly retrieved from Ensembl using biomaRt package (Smedley et al. 2015). Second, we listed all genes in order on basis of lengths of 3′ UTRs and subsequently divided them into four equal parts (also known as the mathematical quarters), and all of which were denoted as gene sets of Q1, Q2, Q3 and Q4, respectively. Accordingly, four equal gene sets (R1, R2, R3 and R4) were also randomly generated independent of 3′ UTR lengths and therefore regarded as the null contrasts. The medians and ranges of 3′ UTR lengths for different gene sets were shown in Table 2. Third, a numerical (*m* × *n*) matrix (*M*) can be organized from both gene annotation of GO terms and these classified gene sets, with *m* representing the number of assigned GO terms and *n* the number of gene sets. The element mij of M denotes the observed frequency for GO term *i* (1, …, *m*) in gene set *j* (1, …, *n*). Finally, principal component analysis (PCA) method, therefore, can be employed to scale differentiation of the GO term-based functional implications of gene sets in matrix *M*, in which the observed frequencies of GO terms were treated as features and the gene set as objects. Based on this design, if the length of 3′ UTRs is not associated with gene functions, the scaling plots of length-based gene sets (Q1, Q2, Q3 and Q4) are expected to be similar to these random sets (R1, R2, R3 and R4).

**Table 2:** Numbers of the retrieved and processed mRNA sequences among four species

Steps	Human	Mouse	Zebrafish	Fruit fly
Raw retrieval	99,884	78,183	47,758	30,246
Prefix of ‘NM_’	39,272	29,686	14,906	30,246
Filtered by length and completeness	36,456	25,420	11,539	26,908
Analyzed mRNA reference sequences	17,351	16,903	11,220	11,661

Additionally, we directly conducted the GO enrichment analysis for these categorized gene sets. Briefly, each gene set was submitted to DAVID system (Huang et al. 2009) and compared against the background of whole genome for obtaining the Functional Annotation Chart. Subsequently, the pairwise comparisons among gene sets based on the Functional Annotation Chart were revealed by CompGO tool (Waardenberg et al. 2015), which employed the log odds ratio scoring (z-score) method for assessing similarities and differences of GO enrichment between experiments.

### Categorization of human genes by tissue and subcellular expressions

Based on a recent publication of human proteome atlas (Uhlén et al. 2015), it is possible to divide human protein-coding genes into different categories according to their patterns of tissue and subcellular expression. Therefore, we utilized these published annotations (http://www.proteinatlas.org) and classified all analyzed human genes in the present study into five known groups of “Tissue enriched” (*N* = 1843), “Group enriched” (*N* = 950), “Tissue enhanced” (*N* = 2907), “Expressed in all” (*N* = 8375) and “Mixed” (*N* = 2300) according to tissue expression patterns, or into four known groups of “Secreted” (*N* = 1779), “Membrane” (*N* = 3543), “Cytoplasm” (*N* = 867) and “Mitochondria” (*N* = 403) by their subcellular localizations. Herein, we directly compared 3′ UTR lengths among these categorized groups.

## Results

### Length distribution of 3′ UTRs

**Fig. 1:**
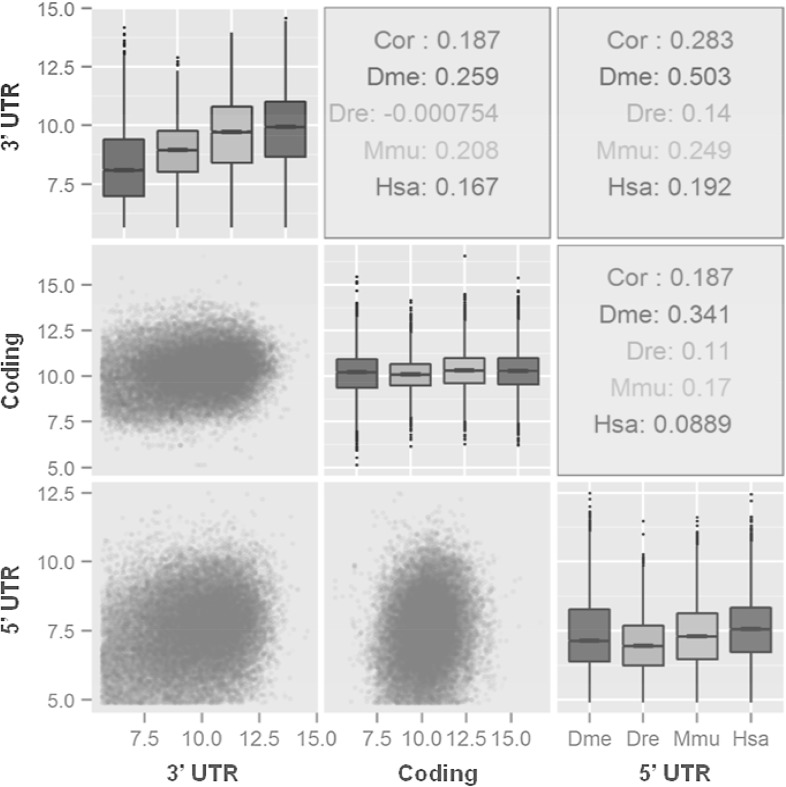
Length distributions and pairwise correlations among 3′ UTRs, coding regions and 5′ UTRs. First, length distributions for 3′ UTRs, coding regions and 5′ UTRs are demonstrated by Box-and-Whisker Plots along the diagonal in the figure, which are also filled by colours for representing different species. Second, the coloured points (lower triangle) graphically demonstrate pairwise comparisons. Third, species-specific Pearson’s coefficients of pairwise correlations are displayed in upper triangle. The lengths of UTRs and coding region (bp) are also log2 transformed for better graphical demonstrations. Throughout all figures, three-letter abbreviations of ‘Dme’ for fruit fly, ‘Dre’ for zebrafish, ‘Mmu’ for mouse and ‘Hsa’ for human are used

**Fig. 2:**
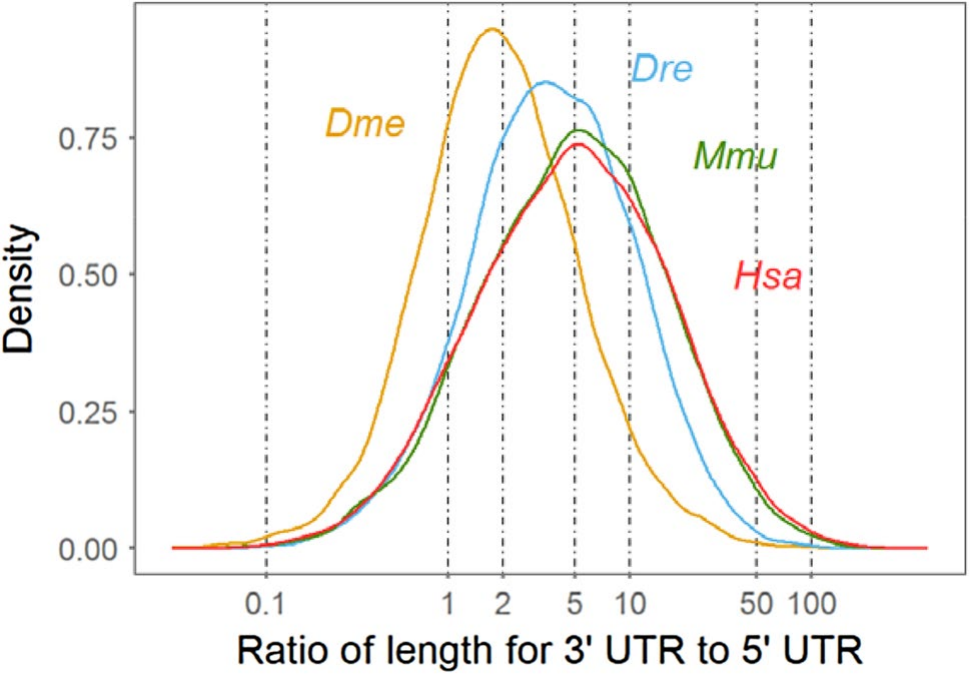
Distributions of relative length of 3′ UTRs to 5′ UTRs for each species

After the custom filtering, a total of 57,135 mRNA reference sequences from fruit fly, zebrafish, mouse and human were finally obtained for investigating 3′ UTR lengths (Table 2). Both the intra- and inter-species comparisons revealed that 3′ UTR lengths are much more variable than that of both 5′ UTRs and coding regions (Fig. 1). It was also clear that 3′ UTR lengths steadily increased from fruit fly to human on the whole, whereas the lengths of coding regions remained almost constant. There was no obvious length correlation for any of the pairwise comparisons among the 3′ UTRs, coding regions and 5′ UTRs (*p* value < 2.2e−16). We further investigated the relative lengths of 3′ UTRs to 5′ UTRs and found that the distribution of length ratio differed significantly among four species (Fig. 2). The 3′ UTR lengths for about 80% of genes in fruit fly were twice as long as 5′ UTRs; this length ratio, however, reached five for about 70% of genes in both mouse and human.

### Evolutionary variation of 3′ UTR lengths

**Fig. 3:**
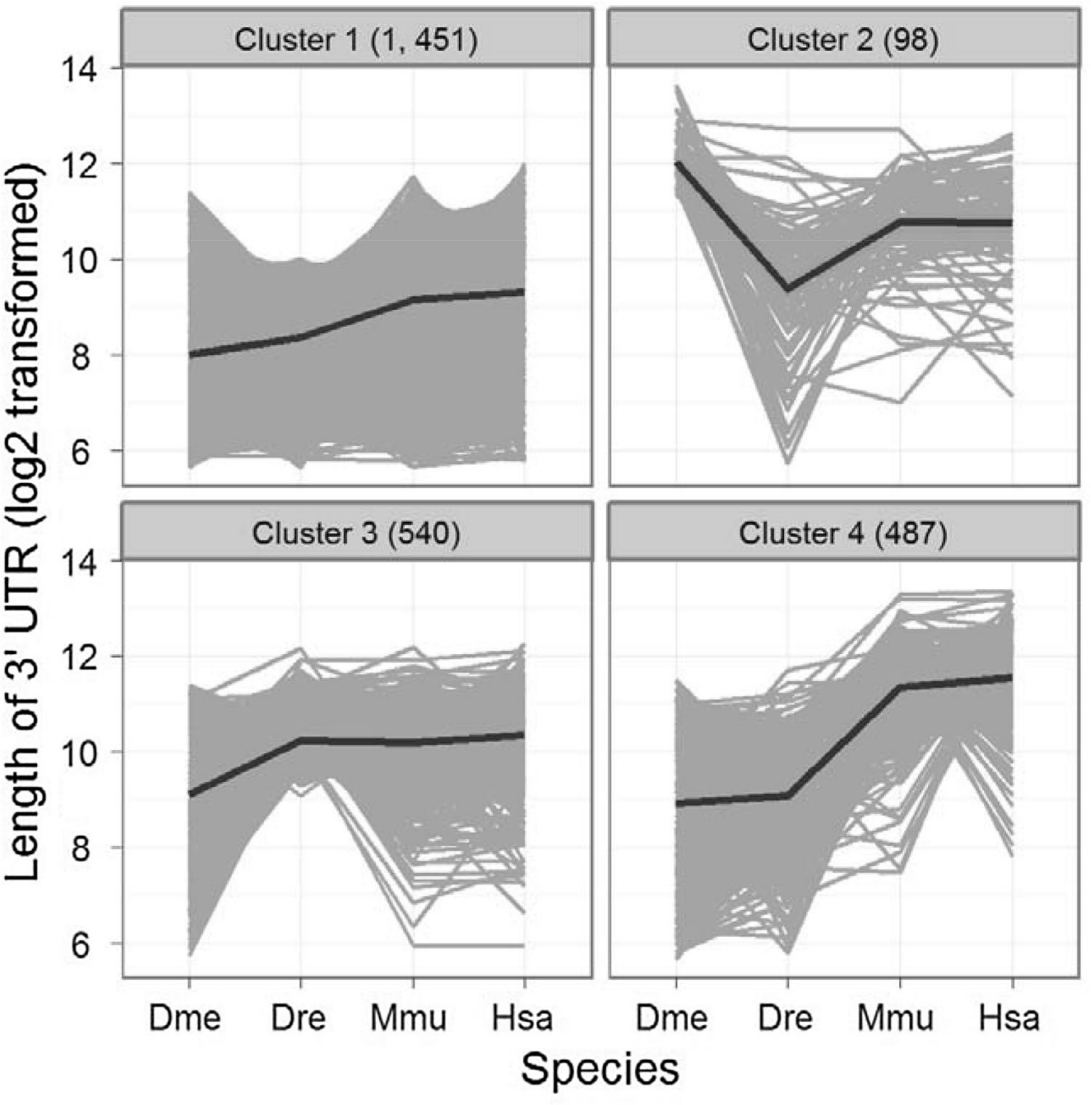
Variation patterns of 3′ UTR lengths for orthologous genes among four species. The gray lines demonstrate all gene-wise variations of 3′ UTR lengths, and black bold lines further denote the cluster centers. The numbers of gene included in each cluster are also shown in brackets

Based on all determined orthologous genes among fruit fly, zebrafish, mouse and human (see Materials and Methods), the k-mean clustering method revealed four obvious clusters corresponding to different evolutionary variation patterns of 3′ UTR lengths from fruit fly to human. Other selections of k-mean parameter were also carefully tested and shown with lower performances by visually inspecting the clustering data. Subsequently, we demonstrated the detailed patterns of 3′ UTR length variations from fruit fly to human for the four clusters (Fig. 3). Among them, Cluster 1 was predominant consisting of 56.3% of genes and showed steady increase in 3′ UTR lengths. By contrast, the increased degrees of 3′ UTR lengths obviously varied from one species to another for both Clusters 3 and 4. Additionally, we also observed an unusual variation pattern of 3′ UTR lengths for genes in Cluster 2, for which the UTRs were longest in fruit fly and shorter in all other species.

### Functional implications of 3′ UTR lengths

**Fig. 4:**
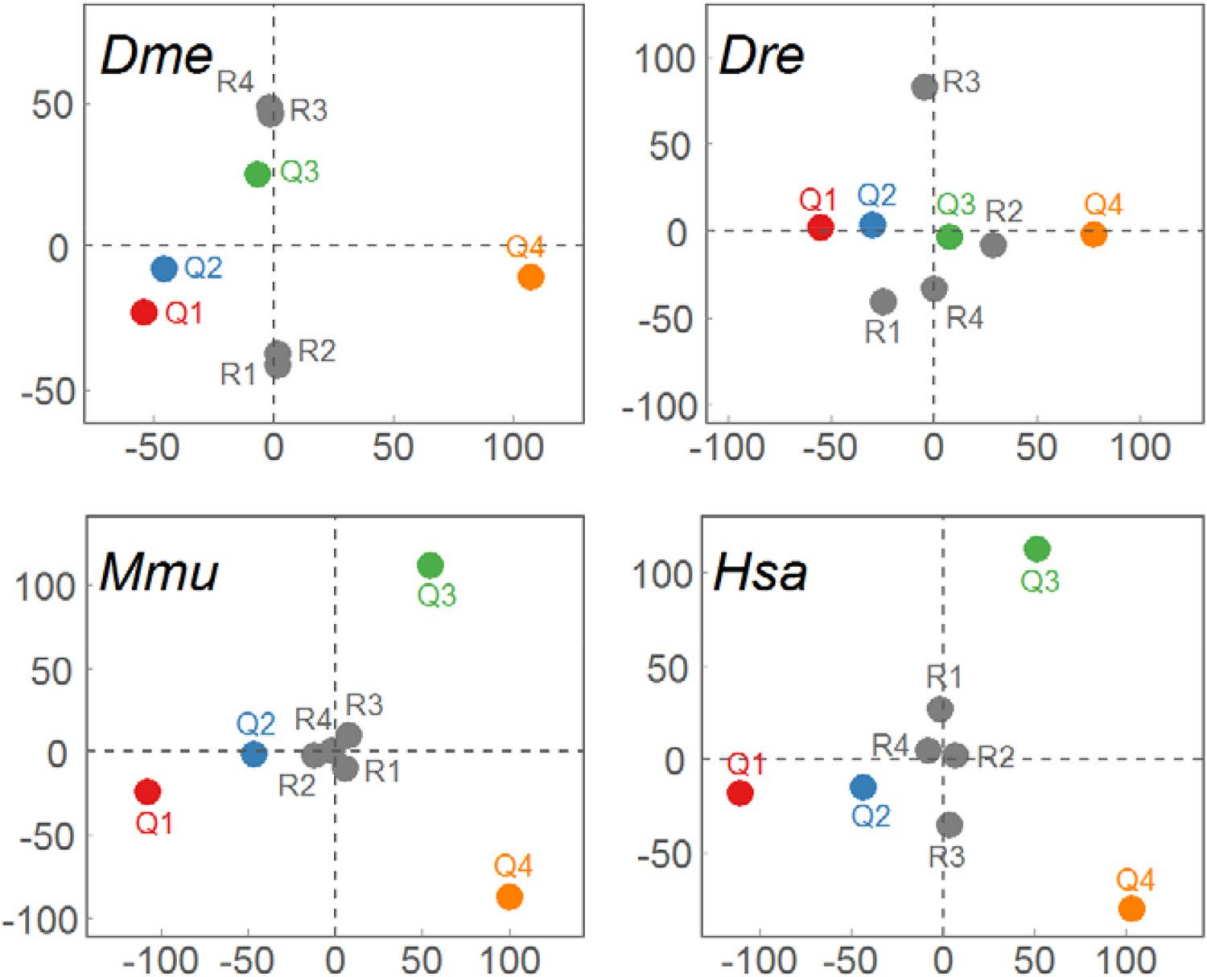
Two-dimensional scaling plots of gene sets according to PCA analyses. The 3′ UTR length-based categories of gene sets (Q1–Q4) are plotted in colour along with their respective labels, whereas the random sets (R1–R4) are in grey. Definitions of different gene sets are detailed in main text

**Fig. 5:**
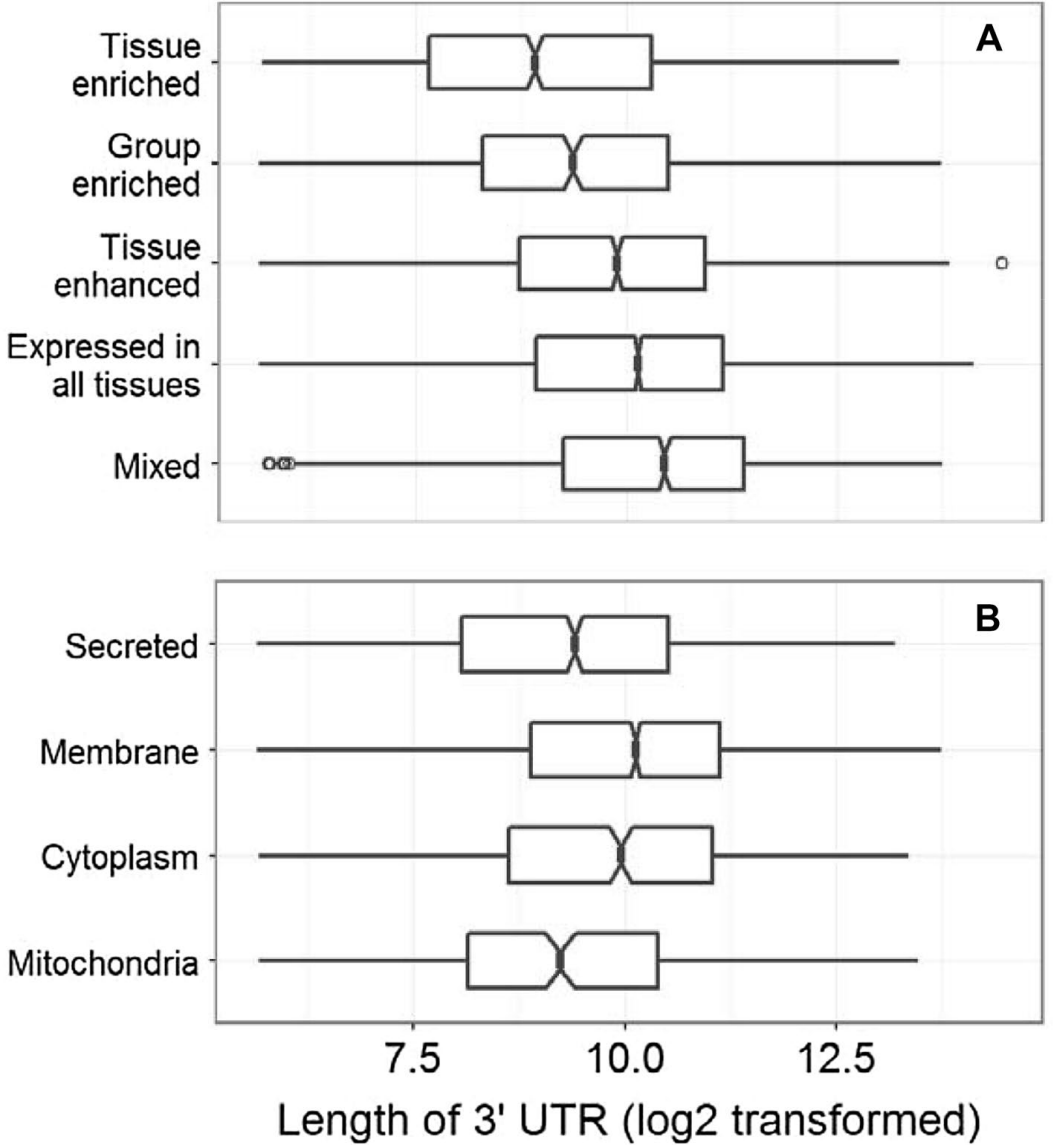
Comparisons of 3′ UTR lengths among different categories of human genes. We categorized five groups according to tissue expression patterns (**a**) or four groups by subcellular localizations (**b**). The notches in boxes represent 95% confidence intervals of median estimations

According to our proposed method for comparing functional implications among various gene sets, we clearly revealed that the 3′ UTR length-based classification of gene sets were scattered with higher differentiation than these observations among randomly generated gene sets in all four species (Fig. 4). However, differences of functional implications were more obvious in both mouse and human than that in both fruit fly and zebrafish. By independently regenerating the random gene sets (R1–R4), we did not observe the obvious changes on their scaling patterns (data not shown). Additionally, we directly investigated the functional similarities by Gene Ontology (GO) enrichment analysis and found no significant correlation for pairwise comparisons among Q1–Q4 gene sets (Supplementary Fig. 1) and also among R1–R4 gene sets (Supplementary Fig. 2) in human. Similar results were also observed for fruit fly, zebrafish and mouse (data not shown). Subsequently, association between the lengths of 3′ UTRs and tissue expression patterns for human genes was investigated. All genes were divided into five groups based on their patterns of tissue expression as reported in a recent human proteome study (Uhlén et al. 2015). The lengths of 3′ UTRs in each of these categories showed differential medians according to the estimations of 95% confidence intervals; and the ‘Mixed’ group had the longest 3′ UTRs. By contrast, there were fewer differences of 3′ UTR lengths among four groups of human genes with different subcellular localizations (Fig. 5). Of course, necessary cautions should be paid to the observed association between the lengths of 3′ UTRs and tissue expression patterns because the statistical significance seems to be some weak.

## Discussion

The sequence length of UTRs is a straightforward feature in eukaryotic mRNAs, which would provide a scaffold for carrying functional elements. However, the length distributions and evolutionary dynamics are observed to be different between 5′ and 3′ UTRs, which are expected to result from differential natural selection (Lynch 2006). In an early report, 5′ UTR lengths were observed to be almost constant across taxonomically diverse organisms with the range between 100 and 200 bp, whereas 3′ UTRs were more variable from a few to thousands of nucleotides in length (Pesole et al. 2001). In the present study, we also revealed that the 3′ UTRs had the greatest intra-species length variation among three regions of mRNAs; however, the intra-species variation for 5′ UTR lengths was also significantly higher than coding regions. Moreover, the inter-species comparisons of length distributions for coding regions remain almost constant from fruit fly to human, which has not been specially addressed yet to our knowledge. In contrast to former report (Liu et al. 2012), we didn’t detect any significant correlation for all pairwise length comparisons among 5′ UTRs, coding regions and 3′ UTRs. Finally, the average length ratio for 3′ UTRs to 5′ UTRs also considerably increased from fruit fly to human; and the formerly proposed conclusion that 3′ UTRs of eukaryotic mRNAs are about twice as long as 5′ UTRs would be biased from analysis on a relative small data set (Pesole et al. 2001).

The overall trend of steadily increased length of 3′ UTRs, which was concluded from sequence analyses among multiple metazoan species (Chen et al. 2012), was clearly observed in 3′ UTRs from fruit fly to human while this pattern was absent for 5′ UTRs. However, we also found that a minority of genes have the longest 3′ UTRs in fruit fly, which should be paid more attentions when deducing the biological meanings. Our comparisons for orthologous gene pairs further revealed that the degree or even direction of 3′ UTR length variations from fruit fly to human obviously varied among individual genes; and by this fact we cautiously speculated that the differential evolutionary patterns of 3′ UTR lengths would be also in relation to their gene functions. Although transcript variants with the shortened 3′ UTRs have been widely observed under pathologic conditions (Mayr and Bartel 2009), it is still unknown whether the genes having different 3′ UTR lengths under normal conditions are associated with divergent biological functions. Based on our straightforward comparisons on the assigned GO terms, we clearly demonstrated that the gene sets as being categorized by 3′ UTR lengths had greater divergences on their functional implications than these random sets; and this result supported a potential correlation between 3′ UTR lengths and gene functions. However, such correlation was not revealed according to the method of GO enrichment analysis because the four random gene sets of R1–R4 even did not show obvious similarities in relation to their functional implications. One possible explanation is that a large number of genes from each set are subjected to the GO enrichments and all of these gene sets don’t have any overlap on their members. Of course, we provided an overall conclusion regarding this topic, which should be subsequently investigated by experimental studies.

In addition to the regulation of translation efficiency, the 3′ UTRs of eukaryotic mRNAs have also been proposed to guide the subcellular localizations and tissue expression patterns. For example, 3′ UTRs were recently demonstrated to determine alternative subcellular expression of CD47 protein on either cell surface or endoplasmic reticulum in human cell lines (Berkovits and Mayr 2015). In human, it was also reported that tissue specific expression of genes may be mediated by differential usage of polyadenylation site (Lianoglou et al. 2013), which is the trigger for generating 3′ UTR isoforms. Uhlén and colleagues (Lynch 2006) published the global landscape of human proteome and classified protein-coding genes into various categories according to subcellular and tissue expression patterns. On basis of these categories, therefore, we compared the 3′ UTR lengths for human genes and found that genes with different tissue expression patterns differed on their median lengths of 3′ UTRs although lacking the statistical conclusion of p-value. On the whole, it seemed that these ubiquitously transcribed genes (such as the groups of ‘Mixed’ and ‘Expressed in all tissues’) had longer 3′ UTRs than these tissue specific genes (such as the group of ‘Tissue enriched’). However, the differences on median lengths of 3′ UTRs were lower among these categories with different subcellular localizations.

Although many mechanisms have been proposed and addressed for the 3′ UTR-mediated post-transcriptional regulations, including alterations on sequence lengths (O’Leary et al. 2015), isoform ratios (Lianoglou et al. 2013), composition of regulatory elements (Oikonomou et al. 2014), and gain-or-loss of microRNA binding targets (Lytle et al. 2007). However, only the measure of 3′ UTR length was included in the present study for comprehensive analyses. A major consideration underlying such design is that 3′ UTR length is a very straightforward feature, which could greatly avoid potential errors or bias and herein facilitate accurate comparisons. In addition, the correlations of 3′ UTR lengths with numbers of regulatory elements and microRNA binding targets had been already specifically investigated in former publication (Chen et al. 2012). Another important character of 3′ UTRs is the secondary structure, which, of course, should not be ignored when accounting for the biological functions. Here, we also provide confident evidences on some general propositions in this field by comprehensive analysis of large-scale data.

## Conclusions

In the present study, we focused on the length of 3′ UTRs and comprehensively analyzed a large-scale highly curated mRNA reference sequences among four well-studied and taxonomically diverse metazoan species. The results provided a landscape of evolutionary patterns of 3′ UTR lengths and correlations with functional implications, which would be helpful for better understanding the 3′ UTRs biology of mRNA in animals.

## Electronic supplementary material

Below is the link to the electronic supplementary material.

Supplementary material 1 (PDF 342 KB)
