# Supplementary material for: Evolutionary and functional implications of 3′ untranslated region length of mRNAs by comprehensive investigation among four taxonomically diverse metazoan species
Source: Genes Genomics. 2019 Mar 21;41(7):747–55. doi: 10.1007/s13258-019-00808-8 (PMC6560010; doi:10.1007/s13258-019-00808-8)

Supplementary Figure 1 Non-thresholded pairwise plot of z-scores among Q1-Q4 gene sets in human. Each circle is a GO term, and the "R" denotes the overall correlation coefficient.

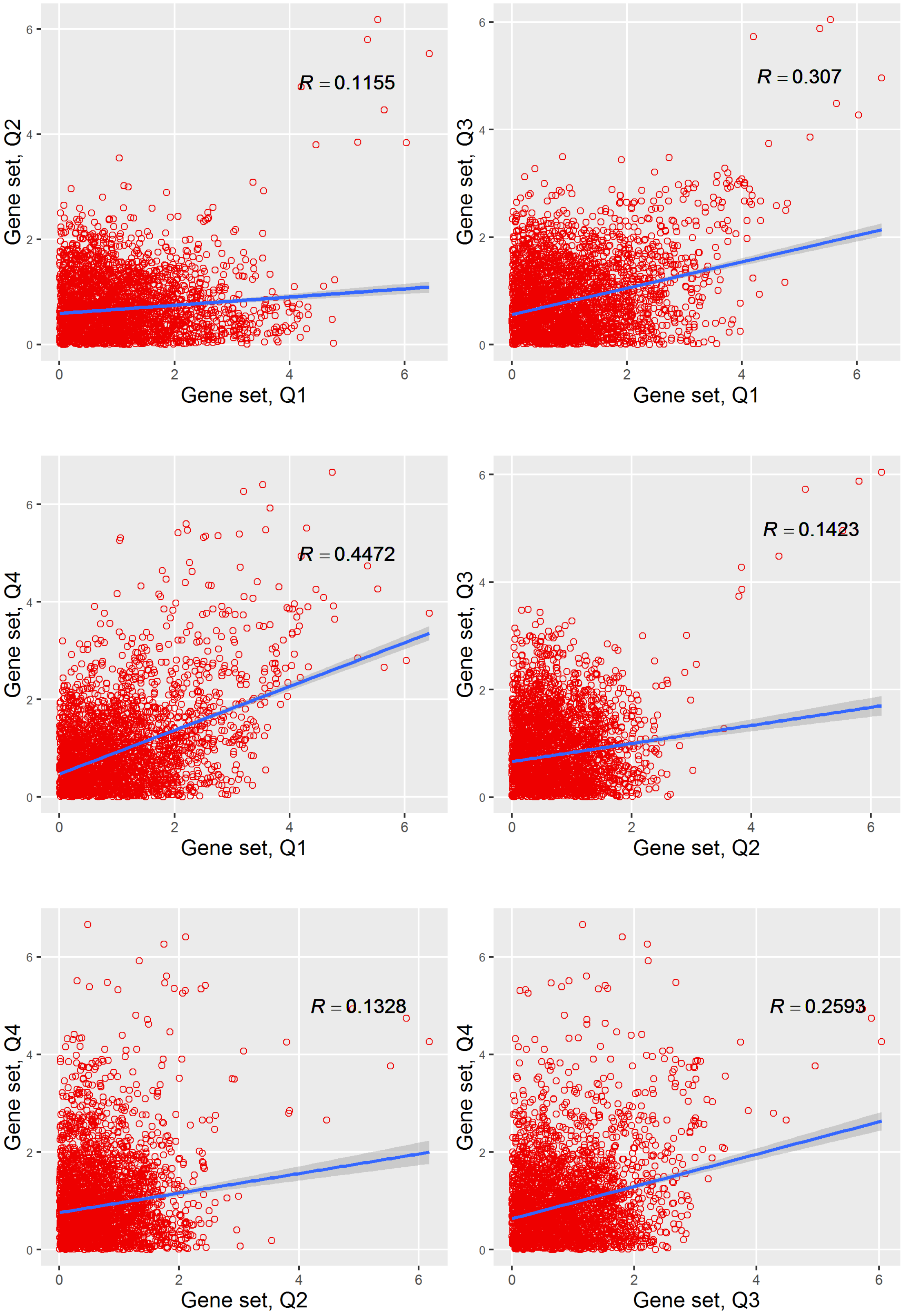

Supplementary Figure 2 Non-thresholded pairwise plot of z-scores among R1-R4 gene sets in human

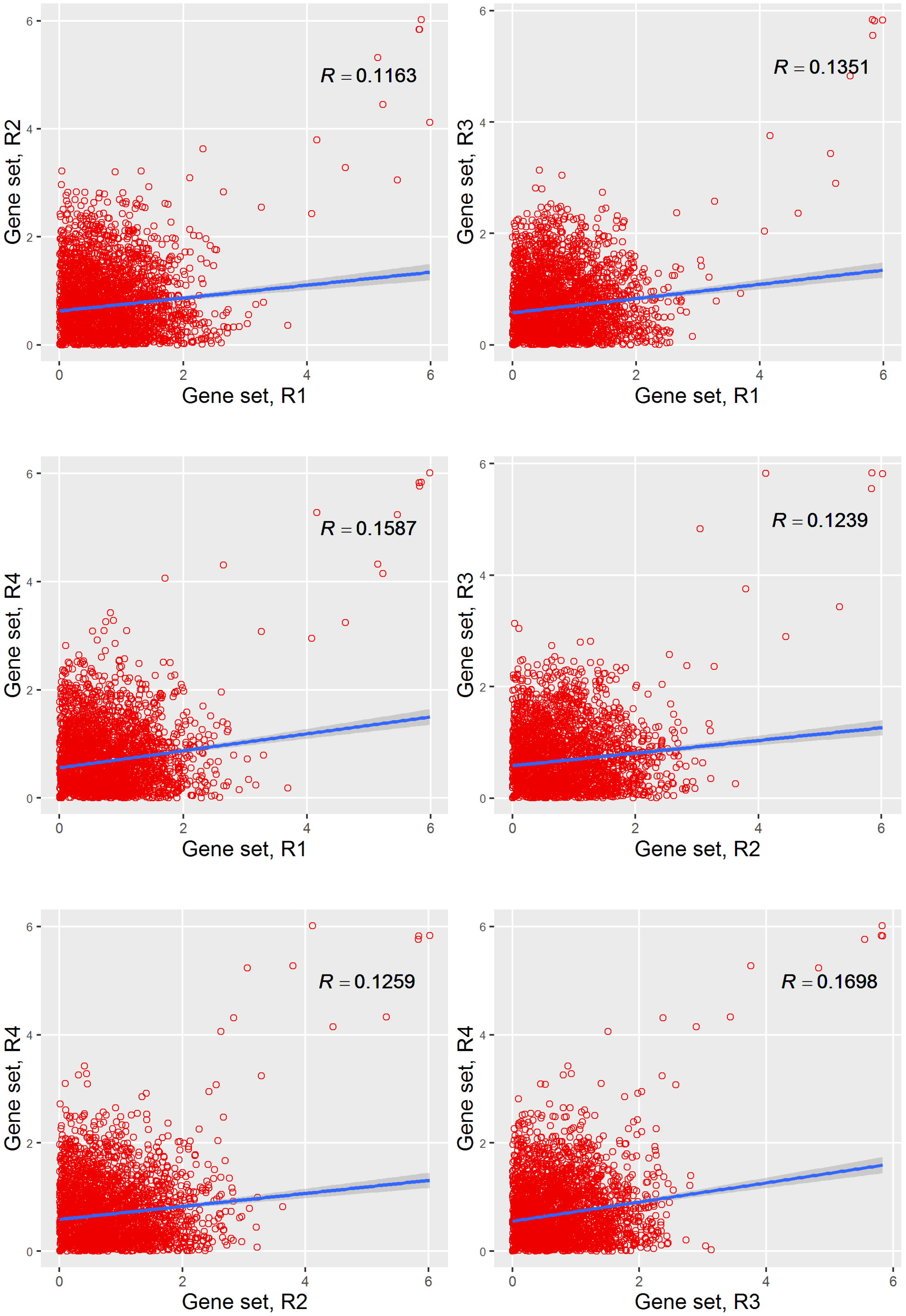

Supplement: Supplementary file 1 — Supplementary material 1 (PDF 342 KB) [file 13258_2019_808_MOESM1_ESM.pdf]
